# Integrated population modelling reveals a perceived source to be a cryptic sink

**DOI:** 10.1111/1365-2656.12481

**Published:** 2016-02-08

**Authors:** Mitch D. Weegman, Stuart Bearhop, Anthony D. Fox, Geoff M. Hilton, Alyn J. Walsh, Jennifer L. McDonald, David J. Hodgson

**Affiliations:** ^1^ Centre for Ecology and ConservationCollege of Life and Environmental SciencesUniversity of ExeterCornwall CampusPenrynTR10 9EZUK; ^2^ Wildfowl & Wetlands TrustSlimbridgeGloucesterGL2 7BTUK; ^3^ Department of BioscienceAarhus UniversityKalø, Grenåvej 14RøndeDK‐8410Denmark; ^4^ National Parks and Wildlife Service, Wexford Wildfowl ReserveNorth SlobWexfordIreland

**Keywords:** capture–mark–recapture, demography, density dependence, dispersal, extinction risk, hierarchical model, long‐distance migrant bird, metapopulation model

## Abstract

1. Demographic links among fragmented populations are commonly studied as source‐sink dynamics, whereby source populations exhibit net recruitment and net emigration, while sinks suffer net mortality but enjoy net immigration. It is commonly assumed that large, persistent aggregations of individuals must be sources, but this ignores the possibility that they are sinks instead, buoyed demographically by immigration.
2. We tested this assumption using Bayesian integrated population modelling of Greenland white‐fronted geese (*Anser albifrons flavirostris*) at their largest wintering site (Wexford, Ireland), combining capture–mark–recapture, census and recruitment data collected from 1982 to 2010. Management for this subspecies occurs largely on wintering areas; thus, study of source‐sink dynamics of discrete regular wintering units provides unprecedented insights into population regulation and enables identification of likely processes influencing population dynamics at Wexford and among 70 other Greenland white‐fronted goose wintering subpopulations.
3. Using results from integrated population modelling, we parameterized an age‐structured population projection matrix to determine the contribution of movement rates (emigration and immigration), recruitment and mortality to the dynamics of the Wexford subpopulation.
4. Survival estimates for juvenile and adult birds at Wexford and adult birds elsewhere fluctuated over the 29‐year study period, but were not identifiably different. However, per capita recruitment rates at Wexford in later years (post‐1995) were identifiably lower than in earlier years (pre‐1995). The observed persistence of the Wexford subpopulation was only possible with high rates of immigration, which exceeded emigration in each year. Thus, despite its apparent stability, Wexford has functioned as a sink over the entire study period.
5. These results demonstrate that even large subpopulations can potentially be sinks, and that movement dynamics (e.g. immigration) among winters can dramatically obscure key processes driving subpopulation size. Further, novel population models which integrate capture–mark–recapture, census and recruitment data are essential to correctly ascribing source‐sink status and accurately informing development of site‐safeguard networks.

## Introduction

The demographic properties of fragmented populations are key to our understanding, conservation and management of biodiversity in the face of habitat degradation and fragmentation. Fragmented populations are formed of subpopulations (Levins 1969, 1970; Fahrig & Paloheimo 1988) that vary in size, stability and dispersal‐mediated connections to the wider population network (Boyce & McDonald 1999; Mackey & Lindenmayer 2001; Manly *et al*. 2002). Census counts of adults and juveniles in large subpopulations can reveal them to be apparently ‘stable’, but classic demographic theory reminds us that short‐term stability can be achieved via imbalances in the processes of survival, recruitment of juveniles, emigration *and* immigration (Pulliam 1988; Watkinson & Sutherland 1995). Here, we use a case study of a long‐distance migrant bird to show that the use of census data risks the misdiagnosis of population persistence because it ignores cryptic demographic processes.

Subpopulations are defined as sources when emigration exceeds immigration and recruitment exceeds mortality and as sinks when immigration exceeds emigration and mortality exceeds recruitment (Pulliam & Danielson 1991; Dias 1996). Sinks can only persist as long as sources (even declining ones) provide immigrants (Watkinson & Sutherland 1995). The apparent stability of any subpopulation might be due either to sustainable levels of recruitment or to bolstering by immigration from elsewhere. Information about rates of birth, death, emigration and immigration is therefore required to properly ascribe source or sink status to subpopulations (Schaub *et al*. 2010); it is naïve to assume that a large, persistent population must be a ‘source’. Indeed, high‐quality (but small) source populations may be critical to the persistence of lower quality (but large) sinks (Doncaster *et al*. 1997). Hence, understanding the internal dynamics of, and connectivity among, populations is critical to understanding, predicting and managing their persistence (Doncaster *et al*. 1997; Hanski & Simberloff 1997; Boughton 1999).

In ecology, source‐sink dynamics have been studied theoretically and demonstrated empirically in a variety of invertebrates, plants and among trophic levels (e.g. between vertebrate and plant communities; for review, see Loreau *et al*. 2013), albeit over relatively short time‐scales. Nonetheless, more comprehensive empirical tests of these dynamics in larger vertebrates are required to better understand how cryptic demographic processes may better inform our perception of sources and sinks, the subject of which has been relatively unstudied. Hence, robust estimation of source‐sink dynamics in animal populations benefits from an integrated modelling approach that combines demographic and census data. With the advent of Bayesian integrated population models (IPMs; Besbeas *et al*. 2002; Schaub *et al*. 2007; Kéry & Schaub 2012), we are now able to analyse whole‐population dynamics alongside capture–mark–recapture (CMR) data to provide robust inference of demographic processes, including rates of age‐dependent mortality, recruitment and dispersal. Here, we use a long‐term data set of marked individuals to model movement events (i.e. emigration and immigration) explicitly and to understand how these events contribute to source‐sink dynamics.

Our study system is the Greenland white‐fronted goose (*Anser albifrons flavirostris*), whose total population breeds in west Greenland, stages during autumn and spring in Iceland, and winters at over 70 regularly occupied sites across Great Britain and Ireland. This population is particularly well suited for analyses of source‐sink dynamics because individuals are long lived and generally site‐loyal, yet exhibit some connectivity between wintering sites (Wilson *et al*. 1991; Warren *et al*. 1992; Marchi *et al*. 2010). Management of Greenland white‐fronted geese occurs largely on wintering areas; however, no studies have assessed whether and how particular wintering sites function as sources or sinks over time. The global Greenland white‐fronted goose population has fluctuated over the last 30 years, but has declined recently by 38%, from 35 700 birds in 1999 to 22 100 in 2012 (Fox *et al*. 2013). However, during this period, the largest wintering subpopulation at Wexford Slobs, Ireland, has remained stable in number. It is the only stable, large wintering subpopulation and has therefore long been considered a source that feeds smaller, more fluctuating subpopulations elsewhere in Great Britain and Ireland. Thus, understanding the function of Wexford is critical for more informed management of this and other Greenland white‐fronted goose wintering areas.

We use IPM to understand the perceived stability of the Wexford subpopulation in the context of the global population decline. By inferring age‐dependent rates of survival, recruitment, emigration and immigration, we parameterize an age‐structured population projection matrix, with associated uncertainty in demographic rates, and use it to determine the contribution of emigration, immigration, recruitment and mortality to the dynamics of this subpopulation. Specifically, we infer demographic rates to (i) determine whether the Wexford subpopulation is a source or, in fact, a cryptic sink; (ii) consider the impact of blocking emigration and immigration on the subpopulation's persistence; (iii) ask whether Wexford's status as source or sink has changed through time; and (iv) consider correlations among, and density dependence of, the demographic processes that contribute to the status of the Wexford subpopulation.

## Materials and methods

### Study area and data sources

The grasslands, cereal and root crop fields of the Wexford Slobs (52°22′ N, 6°24′ W) in south‐east Ireland constitute the single most important wintering area for Greenland white‐fronted geese, supporting over one‐third of the global population (7000–11 000 birds; Warren *et al*. 1992; Fox *et al*. 1998). From 1983 to 2009, 851 juvenile Greenland white‐fronted geese were caught on baited sites at Wexford using traditional cannon‐netting techniques. Captured birds were individually marked with a metal leg band, white plastic leg band and an orange neck collar (both inscribed with an identical alphanumeric code; see Warren *et al*. 1992). Collar code combinations were visible from up to 800 m using a 20–60× spotting scope. Individual geese were aged (juvenile or adult) at capture by plumage characteristics (presence/absence of white frons on face and black belly bars; Cramp & Simmons 1977) and sexed by cloacal examination (Warren *et al*. 1992). A.J.W. resighted geese weekly throughout winter at Wexford, beginning when birds arrived in autumn. A network of volunteers resighted birds annually at over 70 other known wintering sites across Great Britain and Ireland. Among all wintering sites, over 21 400 resightings of known‐age marked birds were recorded during the study period. The total numbers of adults and juveniles at Wexford during winter were estimated by counts and age ratio samples annually from 1983 to 2010. These winter estimates of juveniles in Ireland therefore postdate mortality during hatching, brood‐rearing, fledging and migration prior to the winter census. Thus, we obtained three types of demographic data that contain information about the population dynamics at Wexford: annual adult subpopulation estimates, recruitment at Wexford (the total number of juveniles) and age‐specific (juvenile and adult) survival and movement to and from Wexford and elsewhere. Previous analyses suggest an even sex ratio (Weegman *et al*. 2015); thus, we halved census‐based estimates of adult and juvenile abundances and based our modelling framework on females only.

### Demography of the system

**Figure 1:**
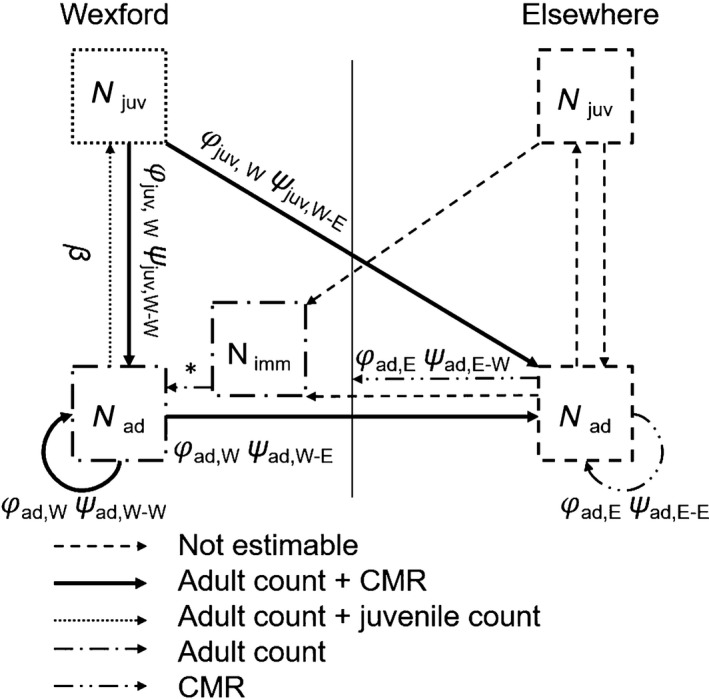
The population processes of Greenland white‐fronted geese wintering at Wexford and elsewhere. The demographic processes occurring at Wexford (to the left of the thin vertical solid line) form the core population model in our IPM. Due to no birds being marked elsewhere and incomplete recruitment data, recruitment and juvenile survival were not estimable elsewhere. The remaining demographic parameters were estimated within the model framework with some parameters common to different sources of information. Notations: ϕ, survival probability for juveniles (juv) and adults (ad) at Wexford (W) and elsewhere (E); ψ, movement probability for each age class between W and E; β, juvenile recruitment; N, the number of juvenile, adult and immigrant individuals. All parameters are also time dependent. Note that immigration would not be identifiable within this state‐space model of adult counts without the information provided by integrating other data sources.

We split the global population of Greenland white‐fronted geese into two wintering units, Wexford and ‘elsewhere’; in the following text, the subscripts W and E refer to these units, respectively. The core of the IPM is a population model based on the Wexford subpopulation, which maps demographic rates onto population size. We developed a female‐based, two age‐structured population model with an annual time step. We assumed a pre‐breeding census in all model structures, such that survival was modelled from year *t* to *t *+* *1 and recruitment in year *t*. The structure of the population model is shown in Fig. 1. The expected number of juveniles (1 year old) and adults (2 years and older) at Wexford in each year can be written mathematically as:

$$\begin{array}{cc} & N_{\mathrm{juv},t+1}=\;\beta_{t}N_{\mathrm{ad},t}\\ & N_{\mathrm{ad},t+1}=\;N_{\mathrm{juv},t}\phi_{\mathrm{juv},w,t}\psi_{\mathrm{juv},w-w,t}+\left(N_{\mathrm{ad},t}+N_{\mathrm{imm},t}\right)\phi_{\mathrm{ad},w,t}\psi_{\mathrm{ad},w-w,t}\\ & N_{\mathrm{imm},t+1}=\;\left(N_{\mathrm{juv},t}+N_{\mathrm{ad},t}+N_{\mathrm{imm},t}\right)\omega_{t} \end{array}$$

*N*_juv*,t*_, *N*_ad*,t*_ and *N*_imm*,t*_ are the number of juvenile, adult and immigrant females in year *t*, respectively, β_*t*_ is the number of juveniles that are recruited in year *t* per female, ϕ_*i*,*w*,*t*_ is the annual survival rate between *t* and *t *+* *1 for individuals in age class *i* and at Wexford, ψ_*i*,*w*−*w*,*t*_ is the probability of an individual in age class *i* remaining at Wexford between years *t* and *t *+* *1, alternatively termed one minus the emigration probability ($1\;-\;\psi_{i,W\;-\;E,t}$). Finally, ω_*t*_ is the number of adult female immigrants in year *t* per female in the population in year *t *− 1.

Using the IPM framework, we modelled the logit of survival and movement as a linear function of age and site, with time as a random effect (Kéry & Schaub 2012). The logs of recruitment and immigration were also modelled with random time effects.

### The joint likelihood

Each model has its own likelihood. The CMR data were analysed via a state‐space formulation of a multistate model, which is comprised of a state and an observation process. The state process describes survival (ϕ) and movement (ψ) probabilities for different ages and locations to describe the true progression through states. As geese were marked as juveniles at Wexford, with no consistent marking elsewhere, juvenile survival and movement probabilities elsewhere were not estimable (Fig. 1). Resightings of adult individuals were recorded at both Wexford and elsewhere, contributing to estimates of adult survival and emigration from Wexford (Fig. 1). The observation process links the true states with the observed states by accounting for variable detection probabilities (*p*; see Supporting Information). A categorical distribution was used to estimate the likelihood of CMR data (*m*). This multistate framework contributes to estimates of survival (ϕ), movement (ψ) and recapture (*p*) probabilities, with the multistate likelihood (*L*_MS_) denoted as *L*_MS_(*m* | ϕ, ψ, *p*).

Population counts of juveniles and adults were analysed using a state‐space model, which consists of a series of equations describing the underlying population processes as outlined in Fig. 1. To account for demographic stochasticity, the number of immigrants and juveniles was modelled using a Poisson distribution, which allowed for unbounded integer values. The numbers of adults were modelled using a binomial distribution with values between zero (if no individuals survived) and the maximum number of individuals the year before (if all individuals survived). Linking the underlying state process to the observed population counts of juveniles (*J*) and adults (*A*) via a Poisson distribution accounted for observation error ($\sigma_{J}^{2},\;\sigma_{A}^{2}$). The likelihood of the state‐space model (*L*_SS_) component is $L_{\mathrm{SS}}\left(J,\;A|N_{\mathrm{juv}},N_{\mathrm{ad}},N_{\mathrm{imm}},\;\phi ,\;\psi ,\;\beta ,\;\omega ,\;\sigma_{J}^{2},\;\sigma_{A}^{2}\right)$.

The likelihoods of the three data sets (juvenile counts, adult counts, CMR) have common parameters, as displayed in Fig. 1. Combining these likelihoods provides more information and reveals previously unidentifiable demographic parameters. Population count data contain information about all demographic processes occurring at Wexford. Hence, when provided with additional information from capture histories, immigration can be estimated as the shortfall between abundances based on census and abundances estimated from CMR (Abadi *et al*. 2010b; Fig. 1). The joint likelihood is the product of the two likelihoods:

$$\begin{array}{cc} & L_{\mathrm{IPM}}\left(m,J,A|N_{\mathrm{juv}},N_{\mathrm{ad}},N_{\mathrm{imm}},\phi ,\psi ,\beta ,\omega ,p,\sigma_{J}^{2},\sigma_{A}^{2}\right)=\\ & L_{\mathrm{MS}}\left(m|\phi ,\psi ,p\right)\times L_{\mathrm{SS}}\left(J,A|N_{\mathrm{juv}},N_{\mathrm{ad}},N_{\mathrm{imm}},\phi ,\psi ,\beta ,\omega ,\sigma_{J}^{2},\sigma_{A}^{2}\right) \end{array}$$

and is based on the assumption that the CMR and census data are independent. While it is possible our study violates the assumption of independence because marked individuals may have been included in population count data, this violation is thought to have little effect on parameter estimates (Abadi *et al*. 2010a), especially when marked birds form a small subset of the total subpopulation. Abadi *et al*. found robust results even when approximately 75% of individuals were included in census and CMR data sets. Individuals in our CMR data set represent approximately only 6% of those counted in the census through time. Abadi *et al*. also discuss simulations where time‐dependent IPMs (such as our own) remain robust to nonindependence of data sets; thus, we do not suspect that a violation of the independence assumption biased our results. Although there exists no goodness‐of‐fit test for IPMs, Schaub *et al*. (2006) and Abadi *et al*. (2010b) recommend assessing fit of component data sets; our goodness‐of‐fit tests on the CMR model suggested no lack of fit.

### Model fitting

We developed Bayesian IPMs using winbugs, version 1.4.3 (Lunn *et al*. 2000; Spiegelhalter *et al*. 2007), adapting examples outlined in Kéry & Schaub (2012). All models were run using the r2winbugs package (Sturtz, Ligges & Gelman 2005) in Program r, version 2.14.2 (R Development Core Team 2012). We used noninformative priors for parameter estimation. Posterior summaries from three Markov chain Monte Carlo (MCMC) chains were based on 160 000 iterations after a burn‐in of 40 000 and a thinning interval of 10. We confirmed model convergence using the Gelman–Rubin statistic (Gelman & Rubin 1992), and >4000 samples were drawn from the posterior distribution with minimal autocorrelation.

We used a series of correlations between demographic parameters to understand the observed stability and to determine whether the Wexford subpopulation was a source (i.e. net exporter) or sink (i.e. net importer from sources) and report all relevant correlation coefficients with 95% credible intervals (CRI). We also report the probability of a correlation lying above or below zero and claim identifiability when this probability is <0·025 or >0·975.

### Population projection matrix modelling

We formed post hoc population projection matrices (Carslake, Townley & Hodgson 2009; Stott, Townley & Hodgson 2011) for the Wexford subpopulation using the R package popdemo (Stott, Hodgson & Townley 2012). Accounting for uncertainty in demographic estimates by using the posterior distributions for recruitment, immigration, age‐specific survival and age‐specific emigration from the IPM, we predicted the asymptotic rate of increase in population growth rate (λ, the dominant eigenvalue of the projection matrix) for three scenarios, where: (i) all demographic parameters were included, (ii) immigration was removed (i.e. ω* *= 0), and (iii) emigration and immigration were removed (i.e. ψ_*W *– *E*_ = 0 and ω* *= 0). The matrix may be expressed as:

$$\begin{array}{cc} & \begin{array}{ccc} N_{\mathrm{juv},t} & & N_{\mathrm{ad},t} \end{array}\\ \begin{array}{c} N_{\mathrm{juv},t+1}\\ N_{\mathrm{ad},t+1} \end{array} & \left[\begin{array}{cc} 0 & \beta \\ \phi_{\mathrm{juv},W}\left(1-\psi_{\mathrm{juv},W-E}\right)+\omega & \phi_{\mathrm{ad},W}\left(1-\psi_{\mathrm{ad},W-E}\right)+\omega \end{array}\right]. \end{array}$$

We compared the posterior distributions of dominant eigenvalues from each analysis to assess the relative contributions of emigration and immigration to population growth rates. This analysis allowed us to determine whether the perceived persistence of the Wexford subpopulation was driven by its internal dynamics of survival and recruitment or by the immigration of birds from elsewhere.

## Results

### Wexford: source or sink?

**Figure 2:**
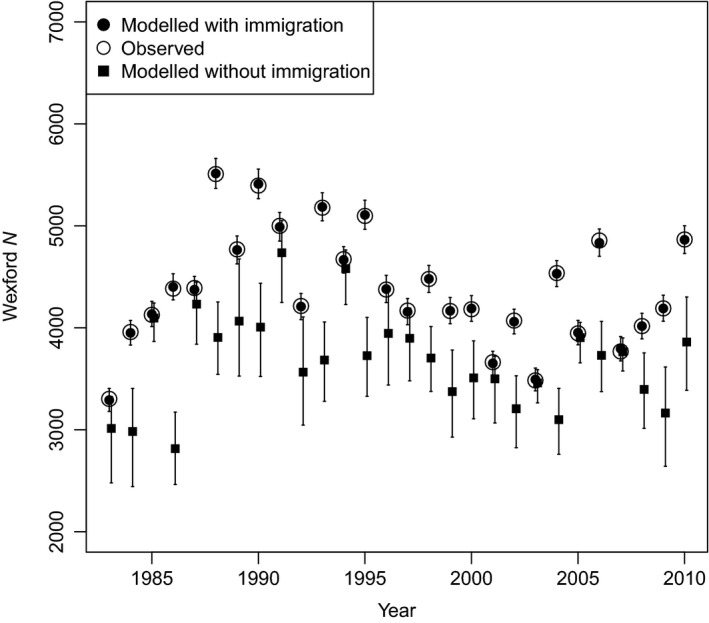
Observed population size and modelled posterior means (with 95% CRI) for Greenland white‐fronted geese at Wexford, Ireland 1983–2010, with (filled circles) and without (filled squares) modelled immigration. When immigration was included in the integrated population model, the observed and modelled population sizes matched (i.e. open circles overlapped filled circles); however, when immigration was not included, the model underestimated population size, demonstrating the reliance of the Wexford subpopulation on immigrants for population stability during the study period.

**Figure 3:**
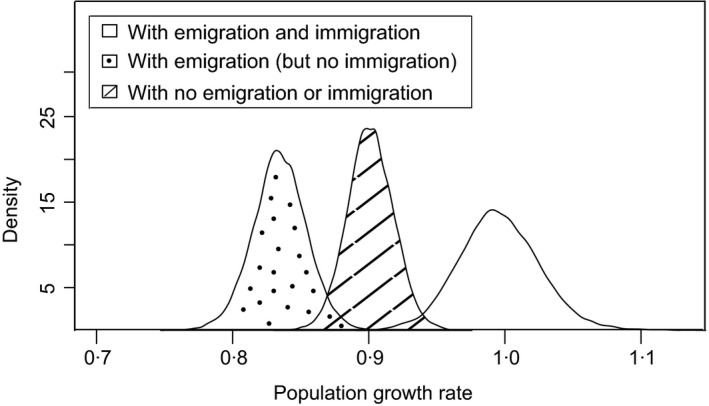
The projected population growth rate when all demographic rates (i.e. emigration, immigration and survival) were included (white), when emigration but not immigration (dotted) was included, and when neither emigration nor immigration (hashed) was included for the Greenland white‐fronted goose subpopulation at Wexford, 1983–2010. Population persistence requires population growth rates near one (i.e. the white distribution). This fig. confirms Wexford is functionally a large sink, where annual immigration is necessary for observed population stability.

Fundamentally, Wexford can only act as a source if it can sustain net emigration of individuals based on net growth due to survival and recruitment. Estimated subpopulation sizes only resembled observed abundances when immigration was inferred (Fig. 2); estimates based only on CMR data resulted in rapid subpopulation decline. This reliance on immigration was confirmed by the posterior distributions of λ, the asymptotic rate of increase of the population projection matrix (Fig. 3). In the presence of age‐structured emigration and immigration of adults, projected λ based on mean demographic rates was 0·99 (95% CRI 0·94–1·06). Blocking migration to and from Wexford yielded a population growth rate of 0·90 per annum (95% CRI 0·87–0·93). Thus, the population cannot sustain itself based on its internal demographic rates. When observed levels of emigration were permitted, the population growth rate dropped further to 0·83 (95% CRI 0·79–0·87). Therefore, across all study years, Wexford has functioned as a large sink.

### Demographic estimates

**Figure 4:**
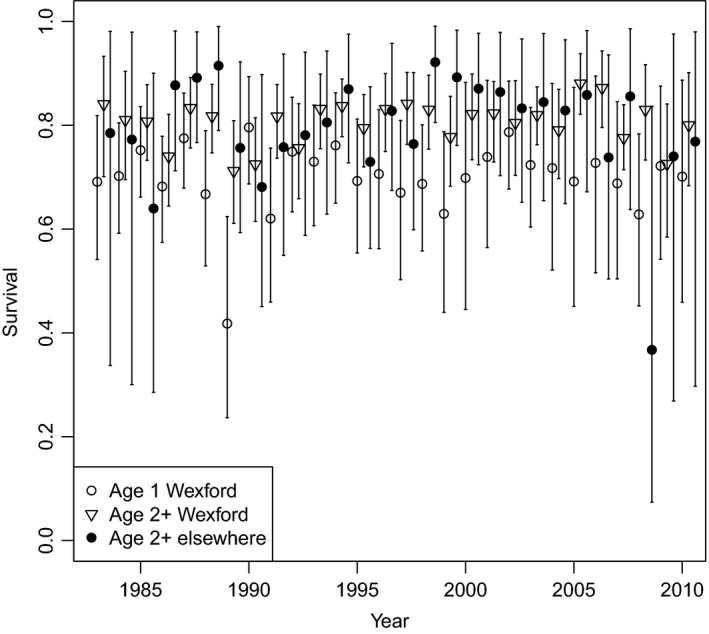
Posterior means (with 95% CRI) for survival of marked Greenland white‐fronted geese aged 1 (open circles) and 2+ (open triangles) at Wexford and age 2+ elsewhere (filled circles), 1983–2010.

**Figure 5:**
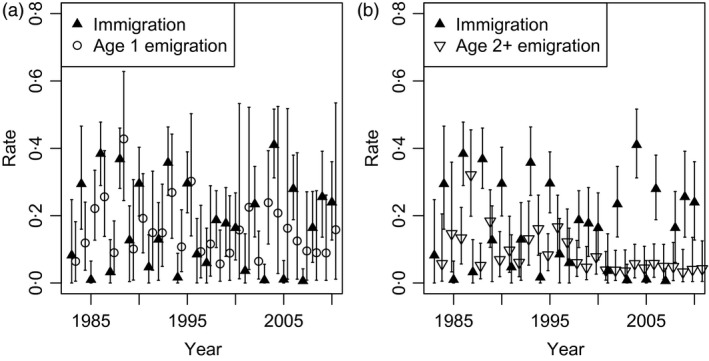
Posterior means (with 95% CRI) of age 1 (open circles; a) and 2+ (open triangles; b) emigration and immigration (filled triangles) in the Greenland white‐fronted goose Wexford subpopulation, 1983–2010.

**Figure 6:**
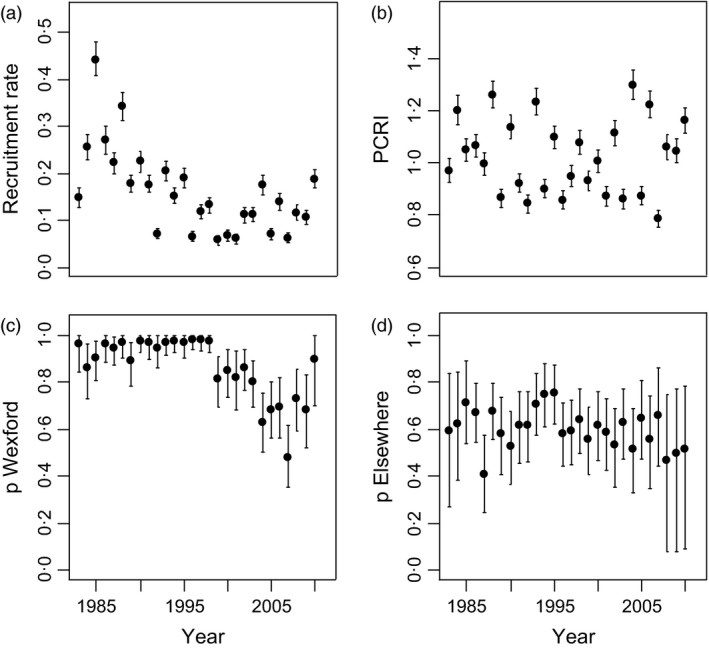
Posterior means (with 95% credible intervals) for recruitment (a) and per capita rate of increase per annum in population size (PCRI; b) in the Greenland white‐fronted goose subpopulation at Wexford, 1983–2010. Resighting probability (*p*) at Wexford (c) and elsewhere (d) were based on geese marked at Wexford, 1983–2010.

Survival of birds aged 1 and 2+ at Wexford and elsewhere was relatively stable over the study period (Fig. 4). For birds aged 1 at Wexford, overall mean annual survival was 0·70 (95% CRI 0·43–0·86). Mean survival of birds aged 2+ at Wexford was 0·81 (95% CRI 0·67–0·91) and of birds aged 2+ elsewhere was 0·79 (95% CRI 0·34–0·98). Thus, adult survival was relatively similar at Wexford and elsewhere. In most years, adult survival was greater than juvenile survival at Wexford. The modelled immigration rate fluctuated over time, from 0·41 in 2004 to 0·01 in 2007 (posterior mean 0·17, 95% CRI 0·01–0·38; Fig. 5a). Further, emigration estimates from Wexford based on multistate CMR models revealed substantial annual emigration for birds aged 1 (posterior mean among years 0·16, 95% CRI 0·01–0·46; Fig. 5a) and 2+ (0·09, 95% CRI 0·01–0·30; Fig. 5b). Resighting probabilities were greater at Wexford (mean among years 0·86, 95% CRI 0·55–0·99) than elsewhere (mean 0·60, 95% CRI 0·31–0·82; Fig. 6c,d). The per capita recruitment rate (juveniles per adult) at Wexford generally declined over the study period, but varied from 0·44 in 1985 to 0·06 in 1999 (Fig. 6a). The per capita rate of increase per annum in population size (*N*_*t + 1*_/*N*_*t*_; hereafter PCRI) also fluctuated between 0·79 in 2007 and 1·3 in 2004, with an average value of 1·02 (Fig. 6b).

### Correlations between PCRI and other demographic rates

The PCRI was strongly positively correlated with immigration rate (*r *=* *0·81, 95% CRI 0·72–0·87; Fig. S1a, Supporting information) and weakly positively correlated with emigration rate for birds aged 1 (*r *=* *0·27, 95% CRI 0·04–0·49; Fig. S1b) and 2+ (*r *=* *0·19, 95% CRI 0·03–0·35; Fig. S1c). PCRI and survival were not significantly correlated (i.e. 95% CRI overlapped zero) for birds aged 1 or 2+ (Fig. S1d,e). However, PCRI and recruitment rate were strongly positively correlated (*r *=* *0·53, 95% CRI 0·49–0·56; Fig. S1f). These results suggest that variation in immigration rate contributed more to variation in PCRI than emigration, survival or recruitment rates.

### Compensatory emigration and immigration

Correlations between emigration and immigration rates were estimated to better understand links between demographic processes at Wexford. The posterior modes of the correlation coefficients (*r*) between immigration and emigration to and from Wexford for birds aged 1 (*r *=* *0·35, 95% CRI 0·07–0·53, *P*(*r*) > 0 = 0·98; Fig. S2a) and 2+ (0·48, 0·28–0·60, *P*(*r*) > 0 = 0·99; Fig. S2b) were identifiable and positive, providing evidence for compensatory processes of population regulation. Recruitment rate was positively correlated with emigration rate of both juveniles (*r *=* *0·40, 95% CRI 0·15–0·59) and adults (*r *=* *0·48, 95% CRI 0·30–0·62), whereby in years of greater recruitment, a greater proportion of birds emigrated (Fig. S2c,d), providing additional evidence of compensatory emigration at Wexford.

## Discussion

Using an IPM which explicitly modelled emigration and immigration from 1983 to 2010, we have shown that the largest and most persistent wintering subpopulation of Greenland white‐fronted geese has only been maintained at observed levels by substantial immigration and has therefore functioned as a cryptic sink. This immigration has resulted in a fluctuating but overall stable population at Wexford over a period of 28 years, despite declines in recruitment, and large increases and decreases in the global population size over the same period. Annual immigration at Wexford was roughly 1400 birds per year (i.e. 17% of the 8000 geese that make up the Wexford subpopulation, assuming an equal sex ratio); further, the Wexford subpopulation relied on immigrants in all but 4 years to maintain the observed stability. Although these findings have been proposed theoretically (Pulliam 1988; Watkinson & Sutherland 1995) and demonstrated in invertebrates and plants (Loreau *et al*. 2013), we consider this to be the first empirical study to show that not all large ‘stable’ subpopulations are sources and that perceived sources can function as cryptic sinks, in which unsustainable internal dynamics are concealed by large‐scale immigration.

Fundamentally, the Wexford subpopulation has functioned as a sink because losses due to mortality have exceeded gains due to recruitment; the population size has only been sustained through immigration. Survival of Greenland white‐fronted geese at Wexford and elsewhere among ages 1 and 2+ was similar to other Arctic‐nesting geese (Schmutz, Rockwell & Petersen 1997; Alisauskas *et al*. 2011; Traylor *et al*. 2012). However, mean recruitment rate (16%) at Wexford was lower than in other Arctic‐nesting geese (Cooke, Lank & Rockwell 1995; Morrissette *et al*. 2010). These estimates assume no marker‐induced mortality associated with neck collars (Alisauskas & Lindberg 2002; Caswell, Alisauskas & Leafloor 2012), which would underestimate true survival and overestimate immigration. However, we contend that any additional mortality due to marker effects would be included in the variance in our estimates and could not account for the large‐scale immigration required to match observed and modelled subpopulations in this study. The large uncertainty in survival and other demographic rates of birds elsewhere was due to relatively few resightings of marked individuals elsewhere; thus, although birds wintering at other sites may have dissimilar demographic rates, we cannot separate this variation (and reduce overall uncertainty in demographics of birds elsewhere) until marking efforts begin at other sites, informing such estimates.

At the global level, threatened species management often focuses on development of site‐safeguard networks protecting large aggregations of individuals (McLean, Wight & Williams 1999) assumed to be sources (Williams, Nichols & Conroy 2002). Often implicit in this focus is an assumption that these subpopulations have self‐sustaining demographic rates. Yet our findings show that the largest concentration of Greenland white‐fronted geese in the world, and one of the few sites which remained approximately stable in number during 15 years of recent global population decline, is only sustained by annual immigration of birds from other (smaller) sites, many of which are in decline, demonstrating the need to understand population dynamics for more informed management of source and sink populations of threatened species.

Our correlations between demographic rates provide evidence of density‐dependent regulation at the main wintering site for these birds, whereby recruitment rate was significantly correlated with emigration rate. This relationship has been demonstrated in other bird populations [e.g. in great tits (*Parus major*; Greenwood, Harvey & Perrins 1979), barn owls (*Tyto alba*; Altwegg *et al*. 2003) and willow ptarmigan (*Lagopus lagopus*; Pedersen *et al*. 2004)] and previous studies on this population (Marchi *et al*. 2010), where immature birds emigrated at greater rates in years when there were proportionally more immature individuals.

Generally, the temporal trends in recruitment and emigration rates declined over the study period. It is unclear what may be causing the low reproductive output observed in recent years, although weather conditions on breeding areas in west Greenland and increasing competition with nesting Canada geese (*Branta canadensis*) have been hypothesized as potential contributing factors (Kristiansen & Jarrett 2002; Boyd & Fox 2008). Declining emigration may be indicative of (i) a larger proportion of the global population at Wexford, where fewer subpopulations may exist elsewhere, (ii) fewer pairs forming as pair bond formation occurs in winter, redistributing birds among subpopulations (Anderson, Rhymer & Rowher 1992) or (iii) longer parent–offspring relationships, which are known to last at least 6 years in this population (Warren *et al*. 1993). If offspring were staying with parents longer, the emigration rate may decrease as older birds are less likely to move permanently between sites (Warren *et al*. 1992). More broadly, the significant positive correlation between emigration and immigration suggests compensatory regulation in the overall Wexford subpopulation size over the study period. This relationship between movement parameters highlights a high degree of connectivity between wintering Greenland white‐fronted goose sites, which has never been quantified and was only previously suspected (Wilson *et al*. 1991; Warren *et al*. 1992).

Our study highlights the importance to wildlife managers of understanding the underlying demographics and source‐sink dynamics for informed management of site networks. It is worrying that cryptic demographic processes can conceal the sink status of apparently stable populations. We suggest complete demographic analyses of source‐sink dynamics should be utilized for more effective designation and management of ‘important’ subpopulations of any species that exist as networks of fragmented populations. Extensions to our demographic analyses could include the marking of individuals across the whole network of subpopulations to improve accuracy of movement estimates and allow the integration of global census data into a source‐sink framework for the whole metapopulation. Fundamentally, we have shown that large aggregations or subpopulations must not be assumed to be sources for smaller subpopulations; cryptic demographic processes like recruitment and dispersal must be considered if we are to understand the impacts of fragmentation on global population dynamics.

## Data accessibility

Source code for all analyses may be found in Supporting information. Data available from the Dryad Digital Repository: http://dx.doi.org/10.5061/dryad.t316t (Weegman *et al*. 2016).

## Supporting information


**Figure S1.** Immigration (a), emigration of geese aged 1 (b) and 2+ (c), survival of geese aged 1 (d) and 2+ (e) and recruitment rate (f) against per capita rate of increase per annum in population size (PCRI) of Greenland white‐fronted geese at Wexford, 1983–2010.

**Figure S2.** Emigration against immigration rate (a,b) and emigration against recruitment rate (c,d) for birds aged 1 and 2+ in the Wexford subpopulation of Greenland white‐fronted geese.

**Appendix S1.** Additional model specification and code.
