# Supplementary material for: Integrated population modelling reveals a perceived source to be a cryptic sink
Source: J Anim Ecol. 2016 Feb 8;85(2):467–75. doi: 10.1111/1365-2656.12481 (PMC4785613; doi:10.1111/1365-2656.12481)

Figure S1. Immigration (a), emigration of geese aged 1 (b) and 2+ (c), survival of geese aged 1 (d) and 2+ (e) and recruitment rate (f) against per capita rate of increase per annum in population size (PCRI) of Greenland white-fronted geese at Wexford, 1983-2010. Black dots show posterior means (with 95% CRI, grey lines). The posterior mode of the correlation coefficients (*r* with 95% CRI) and probability of a positive correlation (P(*r*) > 0) are inset.

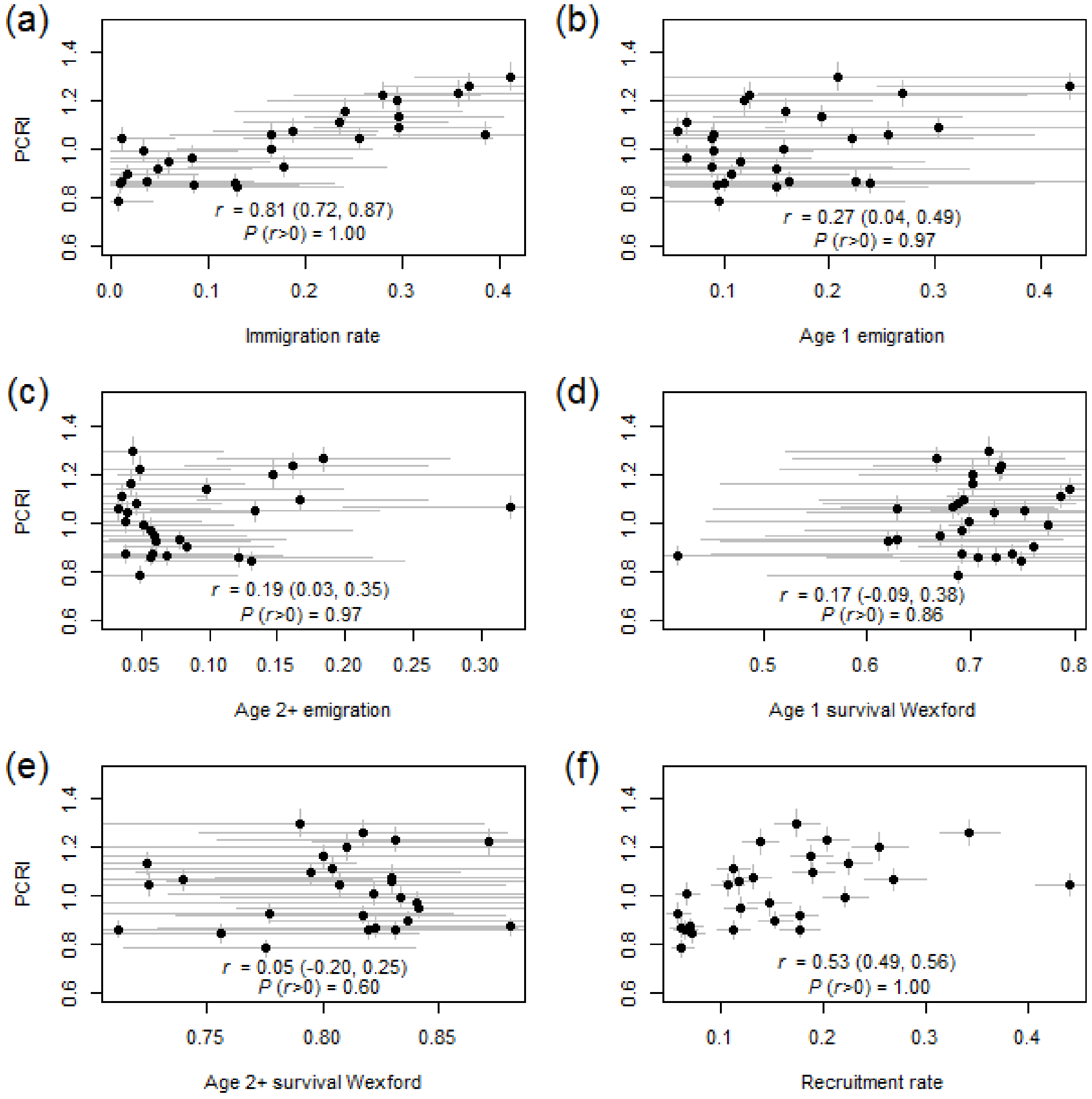

Supplement: Supplementary file 1 — Figure S1. Immigration (a), emigration of geese aged 1 (b) and 2+ (c), survival of geese aged 1 (d) and 2+ (e) and recruitment rate (f) against per capita rate of increase per annum in population size (PCRI) of Greenland white‐fronted geese at Wexford, 1983–2010. [file JANE-85-467-s001.pdf]
