# Supplementary material for: Integrated population modelling reveals a perceived source to be a cryptic sink
Source: J Anim Ecol. 2016 Feb 8;85(2):467–75. doi: 10.1111/1365-2656.12481 (PMC4785613; doi:10.1111/1365-2656.12481)

Figure S2. Emigration against immigration rate (a-b) and emigration against recruitment rate (c-d) for birds aged 1 and 2+ in the Wexford subpopulation of Greenland white-fronted geese. For all figures, black dots show posterior means (with 95% CRI, grey lines). The posterior mode of the correlation coefficients (*r* with 95% CRI) and probability of a positive correlation (P(*r*) > 0) are inset.

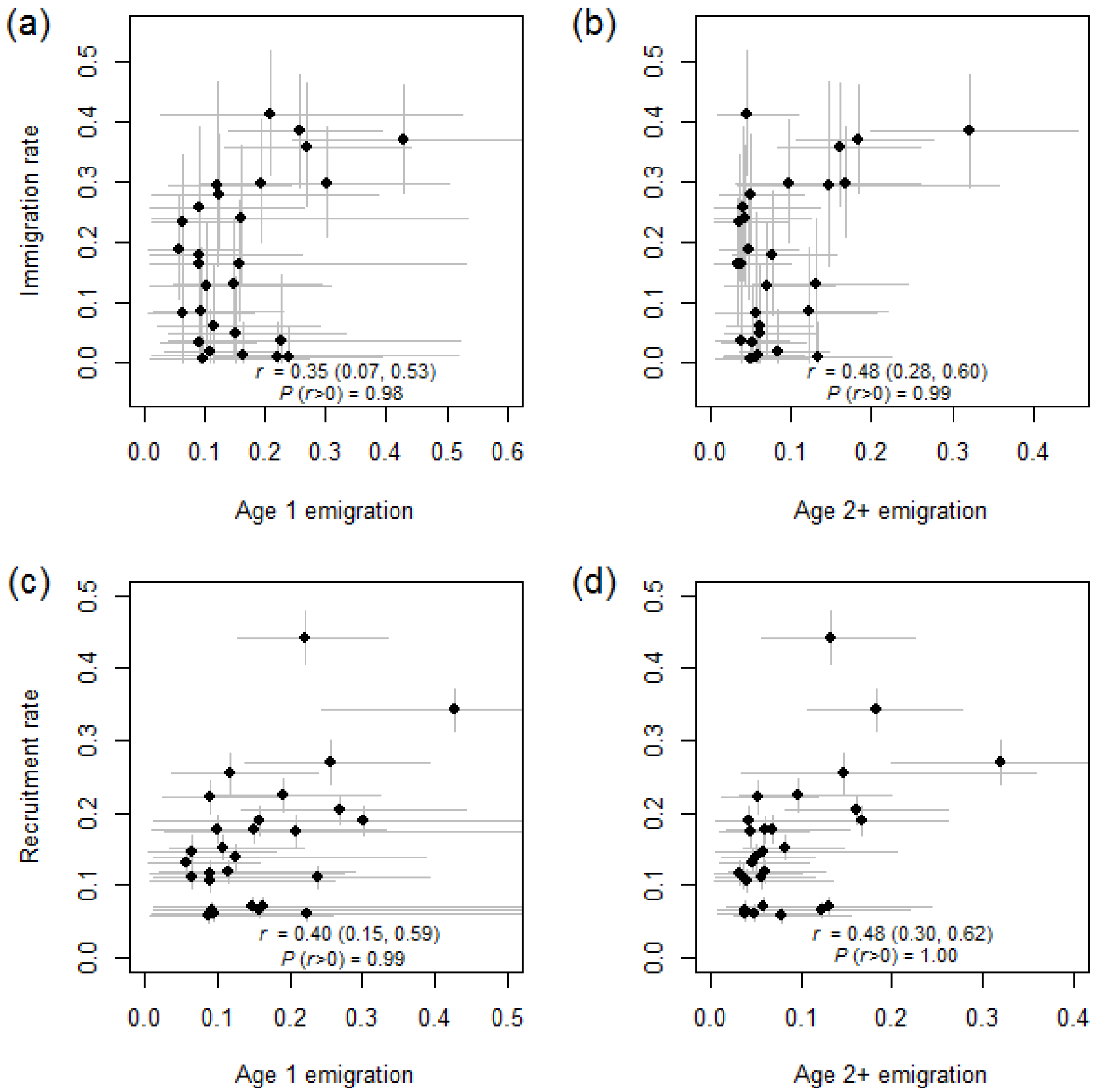

Supplement: Supplementary file 2 — Figure S2. Emigration against immigration rate (a,b) and emigration against recruitment rate (c,d) for birds aged 1 and 2+ in the Wexford subpopulation of Greenland white‐fronted geese. [file JANE-85-467-s002.pdf]
