# Supplementary material for: Integrated population modelling reveals a perceived source to be a cryptic sink
Source: J Anim Ecol. 2016 Feb 8;85(2):467–75. doi: 10.1111/1365-2656.12481 (PMC4785613; doi:10.1111/1365-2656.12481)
Supplement: Supplementary file 3 — Appendix S1. Additional model specification and code. [file JANE-85-467-s003.docx]

**Integrated population modelling reveals a perceived source to be a cryptic sink**

Mitch D. Weegman, Stuart Bearhop, Anthony David Fox, Geoff M. Hilton, Alyn J. Walsh, Jennifer L. McDonald and David J. Hodgson

**Appendix S1, Supporting Information**

**Additional methods**

**State and observation process of the multistate model**

We formed the CMR likelihood using multistate models, which were composed of matrix *m*, with elements *m_i,t_* which indicated the true state of individual *i* at year *t*; three true states were possible (i.e. alive at Wexford, alive elsewhere or dead) and the true state of individual *i* at the time of first encounter was calculated as the vector *fs_i_*. Thus, *fs_i_* was equivalent to the observed state at the first encounter and only events after the first capture were modelled. The state-transition matrix ($\Omega$) was four-dimensional, with dimensions defined by state of departure (*a*), state of arrival (*b*), individual (*i*) and year (*t*). Therefore, the element $\omega_{a,b,i,t}$of $\Omega$ was the probability that individual *i*, which was in state *a* at year *t*, occurred in state *b* at year *t* + 1. The observation matrix (i.e. to calculate resighting probability; $\Theta$) was also four-dimensional, with true state of individual (*a*), observed state of individual (*b*), individual (*i*) and year (*t*). Thus, the element $\phi_{a,b,i,t}$ of $\Theta$was the probability that individual *i*, which was in state *a* at year *t* was observed in state *b* at year *t*. Only two states could be observed (i.e. alive at Wexford or alive elsewhere) because dead individuals in this study were not recovered.

True and observed states were linked by the observation equation, which may be expressed as:

$$n_{i,t}|m_{i,t}\sim categorical(\Theta_{m_{i,t},1...2,i,t})$$

where *n* is the observed multistate CMR data. The state-transition matrix structure for calculation of survival and movement probabilities was based on true states between year *t* and year *t* + 1:

True state at year *t*

Wexford (Wex)

Elsewhere

Elsewhere

Dead

Wex

Dead

True state at year *t* + 1

The observation matrix combined the true and observed states and may be expressed as:

Wex

Elsewhere

Dead

Not seen

True state at year *t*

Observed state at year *t*

Seen at Wex

Seen

Elsewhere

where site-specific time-dependent recapture probabilities were calculated as *p_W_* and *p_E_*. Individuals occurring in a particular site but not observed had probability 1-*p_state_*; for example, the probability that an individual occurred at Wexford but was not observed could be calculated as 1-*p_w_*. For ease of presentation, we have removed some indices in the above matrices. The complete transition and observation matrices include age- and time-dependent survival and transition probabilities, and time-dependent recapture probabilities.

**IPM code**

#-------------------------------------------------

# Load required package

#-------------------------------------------------

**library(R2WinBUGS)**

#-------------------------------------------------

# Specify directory where WinBUGS located

#-------------------------------------------------

**bugs.dir<-"C:/WinBUGS14"**

#-------------------------------------------------

# Load data

#-------------------------------------------------

**census<-read.csv("wexford census.csv",header=T)**

# Convert capture histories to matrix with 'make capture history.r' file

# Number of years

**nyears<-dim(rCH)[2]**

# Number of individuals

**nind<-dim(rCH)[1]**

# Population count data (A = adults)

**A<-c(2583, 2896, 3222, 2708, 3656, 3578, 4290, 4011, 4500, 4206, 3914, 4384, 4005, 4343, 4096, 3686, 3996, 3935, 3922, 3426, 3690, 3076, 4000, 3666, 4349, 3502, 3608, 3808, 4151)**

# Population count data (J = juveniles)

**J<-c(1196, 813, 1473, 2840, 1456, 1624, 2435, 1517, 1791, 1588, 600, 1589, 1337, 1509, 560, 967, 966, 460, 521, 473, 762, 815, 1073, 561, 1015, 531, 819, 764, 1431)**

# Halve juvenile counts to use only females

**J<-round(J/2)**

#-------------------------------------------------

# WinBUGS code to fit IPM. The file is called ‘ipm with movement.bug’

#-------------------------------------------------

**sink("ipm with movement.bug")**

**cat("**

**model {**

# Parameters:

# phiA: survival probability at site Wexford

# phiB: survival probability elsewhere

# psiAB: movement probability from Wexford to elsewhere

# psiBA: movement probability from elsewhere to Wexford

# pA: recapture probability at Wexford

# pB: recapture probability elsewhere

# -------------------------------------------------

# States (S):

# 1 alive at Wexford

# 2 alive elsewhere

# 3 dead

# Observations (O):

# 1 seen at Wexford

# 2 seen elsewhere

# 3 not seen

#-------------------------------------------------

# Define the priors and constraints for the parameters

#-------------------------------------------------

# Initial population sizes

# Set initial population sizes at 1000 individuals, variance 0.0001, capped between # 0 and 3000 (for N1), 0-5000 (for N2old and N2new)

**N1[1] ~ dnorm(1000, 0.0001)I(100,5000) # 1-year**

**N2old[1] ~ dnorm(3000, 0.0001)I(500,5000) # 2-year**

**N2new[1] ~ dnorm(500, 0.0001)I(10,2000) # 2+year**

**N2[1]<-N2old[1]+N2new[1]**

**Nadimm[1] ~ dnorm(1000, 0.0001)I(10,2000)**

**for(a in 1:2){**

**for(u in 1:2){**

**for(t in 1:(nyears-1)){**

**eta.phi[a,u,t]<-mu.phi[a,u]+epsilon.phi[a,u,t]**

**eta.psi[a,u,t]<-mu.psi[a,u]+epsilon.psi[a,u,t]**

**epsilon.phi[a,u,t]~dnorm(0,tau.phi[a,u])**

**epsilon.psi[a,u,t]~dnorm(0,tau.psi[a,u])**

**}** # time

**mu.phi[a,u]~dnorm(0,0.001)I(-5,5)**

**mu.psi[a,u]~dnorm(0,0.001)I(-5,5)**

**sigma.phi[a,u]~dunif(0.1,10)**

**sigma.psi[a,u]~dunif(0.1,10)**

**tau.phi[a,u]<-pow(sigma.phi[a,u],-2)**

**tau.psi[a,u]<-pow(sigma.psi[a,u],-2)**

**sigma2.phi[a,u]<-pow(sigma.phi[a,u],2)**

**sigma2.psi[a,u]<-pow(sigma.psi[a,u],2)**

**}** # site

**}** # age

**for (i in 1:nind){**

# loop through time from first capture

**for (t in f[i]:(nyears-1)){**

# parameters are age- and site-specific

**phiA[i,t] <- 1/(1+1/exp(eta.phi[x[i,t],1,t]))**

**phiB[i,t] <- 1/(1+1/exp(eta.phi[x[i,t],2,t]))**

**psiAB[i,t] <- 1/(1+1/exp(eta.psi[x[i,t],1,t]))**

**psiBA[i,t] <- 1/(1+1/exp(eta.psi[x[i,t],2,t]))**

**pA[i,t] <- 1/(1+1/exp(eta.p[1,t]))**

**pB[i,t] <- 1/(1+1/exp(eta.p[2,t]))**

**}** # time

**}** # ind

**for(u in 1:2){**

**for(t in 1:(nyears-1)){**

**eta.p[u,t]<-mu.p[u]+epsilon.p[u,t]**

**epsilon.p[u,t]~dnorm(0,tau.p[u])**

**}** # t

**mu.p[u]~dnorm(0,0.001)I(-5,5)**

**sigma.p[u]~dunif(0.1,10)**

**tau.p[u]<-pow(sigma.p[u],-2)**

**sigma2.p[u]<-pow(sigma.p[u],2)**

**}** # site

**for (t in 1:nyears){**

**log.mrec[t] ~ dnorm(0,0.001)I(-10,10)**

**log.mim[t] ~ dnorm(0,0.001)I(-10,10)**

**omega[t]<- exp(log.mim[t])**

**mim[t]<-exp(log.mim[t])**

**mrec[t]<-exp(log.mrec[t])**

**}** # time

#-------------------------------------------------

# Specify population-level parameters

#-------------------------------------------------

**for(t in 1:(nyears-1)){**

**psi.age2.site1[t]<-1/(1+1/exp(eta.psi[2,1,t]))**

**psi.age1.site1[t]<-1/(1+1/exp(eta.psi[1,1,t]))**

**psi.age2.site2[t]<-1/(1+1/exp(eta.psi[2,2,t]))**

**phi.age2.site1[t]<-1/(1+1/exp(eta.phi[2,1,t]))**

**phi.age1.site1[t]<-1/(1+1/exp(eta.phi[1,1,t]))**

**phi.age2.site2[t]<-1/(1+1/exp(eta.phi[2,2,t]))**

**p.site1[t]<-1/(1+1/exp(eta.p[1,t]))**

**p.site2[t]<-1/(1+1/exp(eta.p[2,t]))**

**surv.stay.age1.site1[t]<-phi.age1.site1[t]*(1-psi.age1.site1[t])**

**surv.stay.age2.site1[t]<-phi.age2.site1[t]*(1-psi.age2.site1[t])**

**}** # time

#-------------------------------------------------

# Specify derived parameters

#-------------------------------------------------

# Per capita rate of increase per annum in population size

**for (t in 1:(nyears-1)){**

**lambda[t] <- Ntot[t+1] / Ntot[t]**

**}**

#-------------------------------------------------

# Define state-transition and observation matrices that are used in the multistate # model

#-------------------------------------------------

# State transitions

**for (i in 1:nind){**

# Define probabilities of state S(t+1) given S(t)

**for (t in f[i]:(nyears-1)){**

**ps[1,i,t,1] <- phiA[i,t] * (1-psiAB[i,t])**

**ps[1,i,t,2] <- phiA[i,t] * psiAB[i,t]**

**ps[1,i,t,3] <- 1-phiA[i,t]**

**ps[2,i,t,1] <- phiB[i,t] * psiBA[i,t]**

**ps[2,i,t,2] <- phiB[i,t] * (1-psiBA[i,t])**

**ps[2,i,t,3] <- 1-phiB[i,t]**

**ps[3,i,t,1] <- 0**

**ps[3,i,t,2] <- 0**

**ps[3,i,t,3] <- 1**

# Observation

# Define probabilities of O(t) given S(t)

**po[1,i,t,1] <- pA[i,t]**

**po[1,i,t,2] <- 0**

**po[1,i,t,3] <- 1-pA[i,t]**

**po[2,i,t,1] <- 0**

**po[2,i,t,2] <- pB[i,t]**

**po[2,i,t,3] <- 1-pB[i,t]**

**po[3,i,t,1] <- 0**

**po[3,i,t,2] <- 0**

**po[3,i,t,3] <- 1**

**}** # t

**}** # i

#-------------------------------------------------

# Define the integrated population model

#-------------------------------------------------

# Likelihood for juvenile and adult count data (state-space model)

# System process

**for (t in 2:nyears){**

**mean1[t] <- mrec[t-1]*Nad[t-1]**

# Poisson distribution to account for stochasticity of number juvs at t

**N1[t] ~ dpois(mean1[t])**

# Calculate the number of juvenile individuals that survive and remain at Wexford

**N2new[t] ~ dbin(surv.stay.age1.site1[t-1], N1[t-1])**

# Calculate the number of adult individuals that survive and remain at Wexford

**N2old[t] ~ dbin(surv.stay.age2.site1[t-1], Nad[t-1])**

# Calculate total number of adult birds that remain at Wexford

**N2[t] <- N2new[t] + N2old[t]**

# Calculate number of immigrants

**mpo[t]<-Ntot[t-1]*omega[t]**

**Nadimm[t]~dpois(mpo[t])**

**}**

# Observation process - tot adult pop

# Incorporates census data (A and J) with estimates of population sizes (by age # class) and immigration at time t

**for (t in 1:nyears){**

**Nad[t]<- N2[t] + Nadimm[t]**

**Ntot[t] <- N1[t] + Nad[t]**

**A[t] ~ dpois(Nad[t])**

**}**

# Observation process - N1 = J

**for(t in 1:nyears){**

**J[t] ~ dpois(N1[t])**

**}**

# Likelihood of capture-recapture data

# Multistate model 2 age-classes; 2 sites

**for (i in 1:nind){**

# Define latent state at first capture

**z[i,f[i]] <- y[i,f[i]]**

**for (t in (f[i]+1):nyears){**

# State process: draw S(t) given S(t-1)

**z[i,t] ~ dcat(ps[z[i,t-1], i, t-1,])**

# Observation process: draw O(t) given S(t)

**y[i,t] ~ dcat(po[z[i,t], i, t-1,])**

**}** #t

**}** #i

**}**

**",fill = TRUE)**

**sink()**

# End of ‘ipm with movement.bug’

#-------------------------------------------------

# Input into WinBUGS

#-------------------------------------------------

# Function to create known latent states z from Kéry and Schaub (2012)

**known.state.ms <- function(ms, notseen){**

**# notseen: label for ‘not seen’**

**state <- ms**

**state[state==notseen] <- NA**

**for (i in 1:dim(ms)[1]){**

**m <- min(which(!is.na(state[i,])))**

**state[i,m] <- NA**

**}**

**return(state)**

**}**

# Function to create initial values for unknown z from Kéry and Schaub (2012)

**ms.init.z <- function(ch, f){**

**for (i in 1:dim(ch)[1]){ch[i,1:f[i]] <- NA}**

**states <- max(ch, na.rm = TRUE)**

**known.states <- 1:(states-1)**

**v <- which(ch==states)**

**ch[-v] <- NA**

**ch[v] <- sample(known.states, length(v), replace = TRUE)**

**return(ch)**

**}**

# Bundle data

**bugs.data <- list(y = rCH, A = A, J = J, f = f, x=x, nyears = dim(rCH)[2], nind = dim(rCH)[1], z = known.state.ms(rCH, 3))**

# Specify initial values for chains

**inits <- function(){list(log.mrec=rnorm(dim(rCH)[2],-1.5,0.5),log.mim=rnorm(dim(rCH)[2],-1.9,0.5), epsilon.phi=array((0),dim=c(2,2,dim(rCH)[2]-1)),epsilon.psi=array((0),dim=c(2,2,dim(rCH)[2]-1)), mu.phi=array(c(1/(1+exp(-0.71)),1/(1+exp(-0.80)),1/(1+exp(-0.71)),1/(1+exp(-0.81))),dim=c(2,2)),mu.psi=array(c(1/(1+exp(-0.17)),1/(1+exp(-0.10)),1/(1+exp(-0.17)),1/(1+exp(-0.12))),dim=c(2,2)), sigma.phi=array(runif(4, 0.1, 10),dim=c(2,2)),sigma.psi=array(runif(4, 0.1, 10),dim=c(2,2)), epsilon.p=array((0),dim=c(2,dim(rCH)[2]-1)), mu.p=c(rnorm(1, 0.8, 0.01),rnorm(1, 0.3, 0.01)), sigma.p=runif(2,0.1,10), N1 = round(rpois(dim(rCH)[2], 500),0), N2old = round(rpois(dim(rCH)[2], 3000),0), N2new = round(rpois(dim(rCH)[2], 400),0), Nadimm = round(rpois(dim(rCH)[2], 500),0), z = ms.init.z(rCH, f))}**

**inits1<-inits()**

**inits2<-inits()**

**inits3<-inits()**

**initials<-list(inits1,inits2,inits3)**

# Specify which parameters are monitored

**parameters <- c("psi.age2.site1","psi.age1.site1","psi.age2.site2", "phi.age2.site1","phi.age1.site1","phi.age2.site2","surv.stay.age1.site1", "surv.stay.age2.site1","p.site1","p.site2","mu.p","sigma2.p","sigma2.phi","sigma2.psi","N1", "N2","Nadimm", "Ntot", "lambda","mrec", "omega", "mim")**

# Specify MCMC settings: number of iterations, thinning, burn-in and chains

**ni <- 200000**

**nt <- 10**

**nb <- 40000**

**nc <- 3**

# Call WinBUGS from R to run model

**ms <- bugs(bugs.data, inits=initials, parameters, "ipm with movement.bug", n.chains = nc, n.thin = nt, n.iter = ni, n.burnin = nb, debug = TRUE, bugs.directory = bugs.dir, working.directory = getwd())**

**References**

Kéry, M. & Schaub, M. (2012) *Bayesian Population Analysis Using WinBUGS: A Hierarchical Perspective*. Academic Press, Waltham.
